# Sepsis Team Organizational Model to Decrease Mortality for Intra-Abdominal Infections: Is Antibiotic Stewardship Enough?

**DOI:** 10.3390/antibiotics11111460

**Published:** 2022-10-23

**Authors:** Carlo Vallicelli, Giorgia Santandrea, Massimo Sartelli, Federico Coccolini, Luca Ansaloni, Vanni Agnoletti, Francesca Bravi, Fausto Catena

**Affiliations:** 1 General, Emergency and Trauma Surgery Department, Bufalini Hospital, 47521 Cesena, Italy; 2 Department of Surgery, Macerata Hospital, 62100 Macerata, Italy; 3 General, Emergency and Trauma Surgery Department, Pisa University Hospital, 56124 Pisa, Italy; 4 Department of General and Emergency Surgery, Policlinico San Matteo, 27100 Pavia, Italy; 5 Anesthesia, Intensive Care and Trauma Department, Bufalini Hospital, 47521 Cesena, Italy; 6 Healthcare Administration, Santa Maria delle Croci Hospital, 48121 Ravenna, Italy

**Keywords:** sepsis, intra-abdominal infections, sepsis team

## Abstract

*Introduction*. Sepsis is an overwhelming reaction to infection with significant morbidity, requiring urgent interventions in order to improve outcomes. The 2016 Sepsis-3 guidelines modified the previous definitions of sepsis and septic shock, and proposed some specific diagnostic and therapeutic measures to define the use of fluid resuscitation and antibiotics. However, some open issues still exist. *Methods*. A literature research was performed on PubMed and Cochrane using the terms “sepsis” AND “intra-abdominal infections” AND (“antibiotic therapy” OR “antibiotic treatment”). The inclusion criteria were management of intra-abdominal infection (IAI) and effects of antibiotic stewardships programs (ASP) on the outcome of the patients. *Discussion.* Sepsis-3 definitions represent an added value in the understanding of sepsis mechanisms and in the management of the disease. However, some questions are still open, such as the need for an early identification of sepsis. Sepsis management in the context of IAI is particularly challenging and a prompt diagnosis is essential in order to perform a quick treatment (source control and antibiotic treatment). Antibiotic empirical therapy should be based on the kind of infection (community or hospital acquired), local resistances, and patient’s characteristic and *comorbidities*, and should be adjusted or de-escalated as soon as microbiological information is available. Antibiotic Stewardship Programs (ASP) have demonstrated to improve antimicrobial utilization with reduction of infections, emergence of multi-drug resistant bacteria, and costs. Surgeons should not be alone in the management of IAI but ideally inserted in a sepsis team together with anaesthesiologists, medical physicians, pharmacists, and infectious diseases specialists, meeting periodically to reassess the response to the treatment. *Conclusion.* The cornerstones of sepsis management are accurate diagnosis, early resuscitation, effective source control, and timely initiation of appropriate antimicrobial therapy. Current evidence shows that optimizing antibiotic use across surgical specialities is imperative to improve outcomes. Ideally every hospital and every emergency surgery department should aim to provide a sepsis team in order to manage IAI.

## 1. Introduction

Sepsis is a life-threatening organ dysfunction caused by a deregulated host response to infection, according to the Third International Consensus Task Force of 2016 [1]. Severe inflammatory response syndrome (SIRS), sepsis, severe sepsis, and septic shock definitions evolved from the American College of Chest Physicians (ACCP) and Society of Critical Care Medicine of 1991 (SCCM) consensus conferences [2]. Definitions were revised in 2001, during the International Sepsis Definition Conference [3]. In 2016 the Journal of American Medical Association (JAMA) published new criteria for sepsis diagnosis, the so called Sepsis-3 [1]. Finally in 2017, the Global Alliance for Infections in Surgery set up a task-force made of 76 experts from 50 different nations to evaluate the clinical impact of Sepsis-3 definitions. Sepsis-3 definitions are an important step forward in understanding sepsis evolution, and also in distinguishing septic shock from non-complicated infection. However, some years after its publication, some open issues still exist. Intra-abdominal infections (IAI) are known to be associated with a higher complication rate. Particularly, high morbidity and mortality, prolonged hospitalization, and increased hospital costs have been associated with IAI. Patients affected with IAI may be at an increased risk of multi-drug resistant bacterial infections, especially in the case of recent hospitalization or recent surgery. Interestingly, a rate of up to 47% of inappropriate antibiotic prescriptions has been documented in surgical specialties [4]. Infections caused by multi-drug-resistant bacteria are responsible for increased morbidity, prolonged hospitalizations, and higher healthcare costs. Appropriate antibiotic treatment is therefore of the utmost importance and requires frequent re-assessments in the context of ward rounds and antibiotic stewardship programs (ASP). ASP have already been evaluated in literature and their impact on the outcome of the patients in intra-abdominal infections (IAI) has been strongly documented. More recently, the concept of the sepsis team has emerged. The sepsis team is on call and activated at the arrival of the septic patient at the Emergency Department. The sepsis team is in charge of the therapeutic measures undertaken in the emergency setting and during the hospitalization of the patient. The aim of this study was to perform a review of the literature in order to analyze the impact of the establishment of a structured sepsis team for the management of IAI in the emergency general surgery (EGS) setting.

## 2. Methods

A literature search was performed on PubMed and Cochrane to identify suitable publications using the following search terms: “sepsis” AND “intra-abdominal infections” AND (“antibiotic therapy” OR “antibiotic treatment”). The searches were limited to papers fully published in English. The resulting outputs were combined, excluding duplicate results. Abstracts were scanned for suitability and the full text retrieved for all potentially relevant studies. The inclusion criteria for the studies were (1) management of IAI and (2) effects of antibiotic stewardships on the outcome of IAI.

## 3. Sepsis and Septic Shock: Definitions and Controversies

According to the Sepsis-3 definition, sepsis is a life-threatening organ dysfunction due to a deregulated host response to infection, with an increase of Sequential Organ Failure Assessment (SOFA) score of at least two points. Instead, septic shock is defined as a sepsis-induced persistent hypotension requiring vasopressors to maintain a mean arterial pressure (MAP) of over 65 mmHg or having a lactate level over 2 mmol/L despite adequate volume resuscitation (Table 1). Septic shock carries a 40% in-hospital mortality [1].

The process which led to Sepsis-3 definitions involved sepsis concept discussion, the possibility to identify clinical warnings in patients at risk of sepsis, and the development of criteria to identify septic shock. Sepsis definition criteria were processed by scanning a University of Pittsburgh Medical Center-wide database, including 148,907 patients with suspected infection. SOFA score was as valid as Logistic Organ Disfunction Score (LODS) and superior to SIRS in predicting sepsis mortality in Intensive Care Unit [5]. The task-force introduced the quick SOFA (qSOFA) score to identify patients at risk of sepsis but not hospitalized in the Intensive Care Unit (ICU). Sepsis-3 suggests that patients with at least two of the following should be considered at risk of sepsis: (1) hypotension (sistolic blood pressure < 100 mmHg), (2) tachypnea (>22 respiratory act per minute), and (3) alteration of the state of consciousness (Glasgow Coma Scale < 15). Therefore, qSOFA can be useful outside the ICU setting to identify patients at risk of sepsis, but should not be considered a diagnostic criterion of sepsis [5].

However, Sepsis-3 definitions leave some unsolved problems. Indeed, the definition of sepsis as an organ dysfunction appears difficult to apply in the early identification and treatment of the disease. Ideally, from a clinical perspective, patients at risk of sepsis should be identified before the organ dysfunction is evident [6]. Prompt and timely diagnosis and source control are basic principles in managing septic patients and should be performed early [7,8,9,10].

Furthermore the severe sepsis concept is now included in the concept of sepsis itself, even if they are two different conditions with different outcomes [11]. Another critical issue is that Sepsis-3 definition excludes patients with isolated hypotension, who have a SOFA score of 1. Moreover, lactate level is not comprised in the SOFA score, even if it is a reliable marker of severity in patients with an active infectious disease. Difficulties in SOFA standardization outside the ICU setting suggested the task force to introduce qSOFA, which has a poor sensibility [12,13,14,15] and could lead to a high number of false negative with a further diagnostic delay.

## 4. Sepsis Team: The Role in Intra-Abdominal Infection and Emergency General Surgery

Complicated intra-abdominal infections (IAI) are a major cause of morbidity and mortality, especially if poorly managed. The main factors conditioning an effective treatment of IAI infections are a prompt diagnosis, an adequate resuscitation, an early initiation of antimicrobial therapy, an early and effective source control and frequent reassessments of the clinical response of the patient in order to eventually change the management strategy [16].

A prompt diagnosis of IAI should include clinical evaluation, laboratory tests, and imaging (abdominal ultrasound or CT scan). Concerning laboratory tests, procalcitonine (PCT) evaluation can help in the decision of starting antibiotic empirical treatment. PCT level measurement on hospital admission can reduce the initiation of antibiotic therapy in low-risk situations. In high-risk patients, PCT levels can help reduce the duration of antibiotic therapy and guide the decision on antibiotic de-escalation [17,18,19,20]. The source of infection should be adequately controlled as soon as possible. The main goals of the intervention in IAI include determining the cause of the IAI, controlling the origin of the intra-abdominal sepsis with drainage of abscesses or infected fluid collections or tissues, and definitive control of contamination source [21].

Microbiologic evaluation should be performed before starting antibiotic therapy. Blood cultures together with cultures from the site of infection should be obtained whenever possible, and are of utmost importance especially in patients with previous antibiotic exposure [21]. In the literature, there is no clear evidence of utility in collecting cultures from site of infection, because most of the studies concentrate on IAI caused by acute appendicitis, while the benefit of intra-abdominal cultures in patients with IAI caused by conditions other than appendicitis remains unknown [22]. Several guidelines for the management of IAI discussed the role of intra-abdominal cultures and susceptibility testing [16,21,23]. Intra-abdominal cultures are recommended in high-risk patients and those with healthcare-associated IAI. There are no clear recommendations in low-risk patients with community-acquired IAI. An association between taking intra-abdominal cultures and lower mortality in patients with IAI has been reported [24]. If the patient is deteriorating, clinicians should consider upgrade the antimicrobial treatment, and peritoneal cultures can guide to pathogen-directed therapy with more favorable outcomes.

Concerning antibiotic therapy, knowledge of regional and local rates of resistance is essential in the decision-making process while beginning antimicrobial empirical therapy. Predicting the pathogens and potential resistance begins by establishing if the infection is community-acquired or healthcare-associated helps discriminating the proper treatment. In case of community-acquired IAI, narrower-spectrum antibiotics are preferable [25]. Instead, for patients with healthcare-associated IAI antibiotics regimens with broader activity spectrum should be preferred. Among Gram-positive bacteria, *enterococci* play a significant role in IAI. Therefore, *enterococci* coverage should always be considered in patients with perforated appendicitis or perforation of small or large bowel [26,27]. *Enterococci* are common opportunistic micro-organisms isolated increasingly from patients with IAI. Observational studies suggest that the treatment failure of patients infected with micro-organisms such as *Enterococcus* spp. results in increased mortality, but there are no consistent opinions on whether timely anti-enterococcal therapy improves outcomes [28,29,30]. Moreover the variable basic physical conditions of patients with IAI and infection sources usually result in diverse incidences of enterococcal infection [25]. The use of additional agents to provide antienterococcal coverage in the management of community-acquired IAI in lower risk patients has been reported as unnecessary. Instead, risk factors including community-acquired and hospital-acquired infections can increase the risk of enterococcal IAI. Thus, there is a rationale for providing empiric antienterococcal coverage in seriously ill patients with community-acquired IAI and hospital-acquired IAI [31,32].

In critically ill patients antimicrobial therapy should be started early by selecting pharmacological agents with penetration to the presumed site of infection. In case of abdominal sepsis, clinicians must be aware of possible alteration of drug pharmacokinetics due to the mechanisms of sepsis. The effectiveness of antibacterial agents in severely ill patients is primarily related to the maintenance of supra-inhibitory concentrations, so multiple-daily dosing or continuous infusions may be appropriate [33,34]. In patients with uncomplicated IAI when the cause of infection can definitely be treated with surgery, post-operative antibiotic therapy is unnecessary if a complete source-control has been reached. This has been demonstrated, for instance, in the case of uncomplicated appendicitis or cholecystitis and recommended in surgical guidelines [16,35,36]. Instead, in the case of complicated IAI when an adequate source control can be applied, a short course of antimicrobial therapy is recommended (3–5 days) [16]. The Short Course Antimicrobial Therapy for Intra-abdominal Infection (STOP-IT) trial enrolled 518 patients with complicated IAI being treated with an adequate source control. No difference was reported in the two groups after a fixed duration of antibiotic therapy (approximately 4 days) as well as after a longer course of antibiotics (approximately 8 days and until after the resolution of physiological abnormalities) [37]. Empiric antimicrobial therapy should be de-escalated as soon as microbiological information are available. Eventually, in patients with persistent or recurrent clinical evidence of IAI after 4 to 7 days of therapy, further diagnostic investigations should be performed [21].

Antibiotic de-escalation (ADE) consists of the reappraisal of antimicrobial therapy as soon as antimicrobial susceptibility testing results are available and can be applied when the clinical effectiveness of antibiotic therapy is achieved. ADE allows minimizing unnecessary exposure to broad-spectrum agents that would promote the development of resistance [38,39]. However, there is no clear consensus on ADE components and definition, and ADE has demonstrated to be applied in only 40–50% of inpatients with bacterial infection [40].

Indeed, increasing physician confidence and compliance with ADE is a cornerstone of Antibiotic Stewardship Programs (ASP). There is a lack of high-quality clinical data investigating the impact of ADE on antimicrobial consumption and emergence of resistance and a multi-disciplinary approach in managing antibiotic treatment of IAI is mandatory [41]. ASP have been demonstrated to improve antimicrobial utilization and to reduce broad-spectrum antimicrobial use, the incidence of infections, and the emergence of multi-drug-resistant bacteria, antimicrobial-related adverse events, and healthcare-associated costs, with no increase in mortality [42,43,44]. ASP interventions are associated with a decrease in either targeted or overall antibiotic use in critical care patients. However, the approach of reducing the use of certain antibiotic classes is associated with a compensatory use in unrestricted antibiotics (“squeezing the balloon” effect). Similarly, after six months, most ASP interventions has been associated with decreased resistance among main ICU microorganisms, but restriction policies have been associated with some decreased susceptibility rates to unrestricted antibiotics. Therefore, active interventions rather than passive ones can be associated with more favorable outcomes. Moreover, the reduction of antimicrobial utilization in the context of ASP does not relate with any worsening in nosocomial infection rates, length of stay, or mortality [42]. Karanika et al. reported an association between the implementation of ASP and a decrease in consumption of high-potential-resistance antimicrobial agents, such as carbapenems and glycopeptides. This is indicative of the fact that the choices on antibiotic regimens were probably more appropriated. Indeed, ASP seem to be effective not only because of a decrease in quantity of antimicrobial consumption, but due to a true positive effect on antibiotic choices. Moreover, the implementation of ASP is associated with a significant drop in antimicrobial costs (more than one third) [43]. Baur et al. observed that implementation of ASP is associated with a reduction in the rates of infection and colonisation with antibiotic-resistant bacteria and *Clostridium* infections in hospitalized patients. The largest reduction was seen in the incidence of infection or colonisation with multi-drug-resistant Gram-negative bacteria, followed by ESBL-producing Gram-negative bacteria, *MRSA*, and *Clostridium Difficile* infections [44].

ASP is defined as a coherent set of actions with the aim of more appropriate antimicrobial use, in order to ensure sustainable access to effective therapy for all who need them [45]. Every ASP should focus on three main kinds of interventions:

- Restrictive: efforts to reduce the number of opportunities for bad behaviors, such as formulary restrictions, approval by a recognized ASP expert doctor, and automatic stop orders;
- Collaborative or enhancement: efforts to increase the numbers of opportunities and decrease barriers for good behavior, for instance with education of prescribers, implementation of treatment guidelines, promotion of ADE, use of pharmacokinetics and pharmacodynamics concepts, prospective audits, and feedback;
- Structural: may include the use of computerized antibiotic decision support systems, faster diagnostic methods for antimicrobial resistance, surveillance systems, and daily collaborations between physicians, pharmacists, nurses, infection control units, and microbiologists [38,46].

ASP have been demonstrated to be of utmost importance in the management of in hospital infection diseases (Table 2).

However, this concept is particularly true in IAI due to the complexity of the disease and of the EGS setting. IAI are associated with high morbidity and mortality rates, prolonged hospitalization, and increased healthcare-related costs. Surgeons often have to face complex clinical and organizational scenarios in emergency general surgery (EGS) and should not be alone when managing antibiotic therapies for IAI. Every EGS department should develop a sepsis team including emergency surgeons, infectious disease specialists, anesthesiologists, pharmacists, and internal medical physicians.

The emergency department (ED) is normally the place where sepsis patients are seen initially. Simon et al. evaluated in-hospital mortality rates of sepsis patients admitted to an urban tertiary care ED before and after the implementation of sepsis teams. In this study, 553 patients were included in the pre-implementation group and 635 patients in the post-implementation group. Among septic patients, the mortality rate was significantly reduced by the implementation of sepsis teams. In this study, a 56% decrease for in-hospital mortality was observed [47]. Girardis et al. evaluated 67 consecutive patients admitted to the ICU because of severe sepsis or septic shock over a 2-year period. Inclusion criteria were documented or suspected infection; two or more SIRS criteria; and onset of an organ dysfunction related to the infection. An in-hospital program dedicated to sepsis was developed, including healthcare personnel education and specific process changes. The sepsis team was available 24 h per day, with a dedicated telephone number, guaranteeing a consultation within 60 min in case of severe sepsis and within 30 min in case of septic shock. The implementation of the sepsis team improved the adherence to guidelines and also the survival rates of the patients [48]. Delawder et al. reported the effect of the implementation of an interdisciplinary sepsis-response team in the ED on improved bundle compliance and mortality. A total of 214 patients admitted with sepsis were included in the analysis. With the implementation of the sepsis team program, mortality rates showed a steady decline from 12.45% to 4.55% [49] (Table 3).

The sepsis team should be in charge of the management of the IAI. The sepsis team should meet at the initial evaluation of the patient and afterwards periodically during ward rounds, ideally every 48 to 72 h in order to reassess the response to the treatment. This organizational model is presumed to improve the outcomes of the patients, and dedicated pathways are required in order to overcome the expected organizational obstacles encountered in the real daily life.

## 5. Emergency Surgeons: The Daily Life

Evidence shows that antimicrobial use recommendations to surgical services have significantly lower odds of acceptance as compared with those made to general medicine providers. Surgeons are known to be reluctant to accept recommendations on antibiotic therapy. Indeed, surgeons have lower odds of agreement concerning de-escalation [50]. Recommendations aiming to decrease antibiotic treatment were less likely to be accepted as compared with those that improve antimicrobial spectrum coverage [51]. Daily life in the EGS department can be particularly complex and this can explain the high rate of reported inadequate antibiotic usage in surgical departments. This situation can be related to the limited time surgeons tend to spend on the wards, due to the operating theater activity. In real daily life, many duties outside the operating theater are delegated to junior surgeons and antibiotics decisions are mostly conducted by them and just approved later on by senior surgeons [4]. Surgeons appear to be afraid of negative patient outcomes that could lead to inappropriate antibiotic use, especially after surgery [44,52,53]. Moreover, comparing surgeons with medical physicians, individualism in surgery influences decision making. The surgical team is usually vertical in structure, with a leader making decisions and having less time or opportunity for team input. On the other hand, in medical wards, a more collectivist culture prevails, and the presence of the pharmacist reinforces the necessity to review patient medications. The surgical team focuses primarily on the “now”, requiring the team to plan and schedule, and has three main commitments: the operating theater, clinics, and the ward. As a consequence, there is a dispersion of time and a disjointed method of work, with less communication compared with the environment of medical teams and a lack of multidisciplinary inputs into patient care [44]. Moreover, in surgery, the diagnosis of infection frequently relies on infection markers (C-reactive protein, leucocytes count) and temperature, and the decision-making process is focused on prevention and prophylaxis rather than on the treatment of the IAI. Instead, in medical settings, infection markers are part of the decision process, but medical team members try to rationalize their decisions. Moreover, medical teams make efforts to align them with local policy and involve and discuss with other healthcare professionals. For these reasons, difficult decisions such as ADE can often be deferred in surgical wards and this may result in prolonged and unnecessary antibiotic course for surgical patients.

Therefore, the strong need for a sepsis team in IAI management appears to be clear. Surgeons are a fundamental part of the sepsis teams but are required to work closely with infectious disease specialists, medical physicians, and anaesthesiologists in order to improve antibiotic therapy management and the outcome of the patients. Frequent reassessment of antibiotic treatments for IAI should be performed in the context of ward rounds. Hospitals and EGS departments should guarantee dedicated pathways for IAI treatment, letting different specialists meet together routinely every 48 to 72 h. On their hand, surgeons are called to accurately review their daily life activity, putting decision on IAI management at the top of their daily schedule.

## 6. Conclusions

Sepsis and septic shock are complex concepts, with complex pathogenesis characterized by interaction between the host immune system and pathogenic microorganism. Clinical manifestations of IAI are extremely various and can eventually lead to organ dysfunction and an increased rate of morbidity and mortality. Early diagnosis and prompt treatment are basic principles and cornerstones of IAI management. Sepsis-3 definitions improved our knowledge and understanding of sepsis pathogenesis, underlining the concept of immune response deregulation leading to organ dysfunction. However, Sepsis-3 could be not effective enough to identify patients before organ dysfunction becomes evident.

The cornerstones of IAI management include early and accurate diagnosis, prompt resuscitation, early and effective source control, and initiation of appropriate antimicrobial therapy. Antibiotic choice, molecular diagnostics, duration of therapy, and optimal dosing is essential to ensure the best therapeutic outcomes. ADE was demonstrated to optimize antibiotic treatment without compromising clinical outcomes. Using the shortest effective course of antibiotic therapy decreases antibiotic pressure that can potentially lead to resistance and the risk of adverse events.

Eventually, the observed variation in social norms, values, and behaviors in medicine and surgery defines the approach to antibiotic decision-making. The medical team adopts a more policy-driven, interdisciplinary approach that includes pharmacist and infectious disease input. Instead, the surgical teams tend to perceive themselves as interventionists and see ASP as has having a low priority. Optimizing antibiotic use across surgical specialities is imperative to improve outcomes. Therefore, ASP should be strongly improved in every EGS department. Ideally, a sepsis team should be established in every hospital to properly manage IAI. The close relationship between surgeons, pharmacists, infectious disease specialists, and anaesthesiologists has been demonstrated to optimize the management of IAI and to improve the outcome of the patients. Therefore, an effort to guarantee dedicated pathways for sepsis team development is strongly advised for EGS departments.

## Figures and Tables

**Table 1:** Evolution of sepsis definition over time.

	Original Sepsis Definitions (ACCP and SCCM 1991 Consensus Conference)	New Sepsis-3 Definitions (2016 Third International Consensus Definitions for Sepsis and Septic Shock)
SIRS	A clinical response arising from a non specific insult, including 2 of the following: - Temperature >38 °C or <36 °C - HR ≥ 90 beats/min - Respirations ≥ 20/min - WBC count ≥ 12,000/mm^3^ or <4000/mm^3^ or >10% immature neutrophils	X
Sepsis	SIRS with a presumed or confirmed infectious process	- Life-threatening organ dysfunction caused by a dysregulated host response to infection - Suspected or documented infection and - Acute increase of ≥2 SOFA score points (proxy for organ dysfunction) - Hospital mortality > 10%
Severe Sepsis	Sepsis with more than one sign of organ failure: - Cardiovascular (refractory hypotension) - Renal - Respiratory - Hepatic - Hematologic - CNS - Unexplained metabolic acidosis	X
Septic Shock	Sepsis-induced hypotension despite adequate fluid resuscitation, with perfusion abnormalities	Sepsis with persistent hypotension: - Requiring vasopressors to maintain MAP ≥ 65 mmHg - Serum lactate level ≥ 2 mmol/L (18 mg/dL) despite adequate fluid resuscitation. - Hospital mortality > 40%

**Table 2:** Major studies available investigating the outcomes of Antibiotic Stewardship Programs (ASP).

Authors, Year	Article Type	Investigation
Timsit JF, et al., 2019 [39]	Narrative review	Implementation of ASP in the ICU to improve antibiotics administration
Tabah A, et al., 2015 [41]	Systematic review	Definition and outcomes of ADE in ICU patients
Kaki R, et al., 2011 [42]	Systematic review	Outcomes of ASP in ICU: improved antimicrobial utilization, improvements in antimicrobial resistance and adverse events without compromise of short-term clinical outcomes.
Karanika S, et al., 2016 [43]	Systematic review and meta-analysis	Hospital ASPs result in significant decreases in antimicrobial consumption and cost, and the benefit is higher in the critical care setting.
Baur D, et al., 2017 [44]	Systematic review and meta-analysis	ASP significantly reduce the incidence of infections and colonization with antibiotic-resistant bacteria and *Clostridium difficile* infections in hospital inpatients.
Dyar OJ, et al., 2017 [45]	Narrative review	Literature’s review on ASP

**Table 3:** Major studies investigating outcomes of implementation of sepsis teams.

Authors, Year	Study Protocol	Outcomes
Simon, et al., 2022 [47]	553 patients pre-implementation vs. 635 patients post-implementation	56% decrease for in-hospital mortality
Girardis, et al., 2009 [48]	67 consecutive patients admitted to ICU with severe sepsis or septic shock	Improved survival rates
Delawder, et al., 2020 [49]	214 patients admitted to ED with sepsis	Mortality rates showed a steady decline from 12.45% to 4.55%
